# Painless Dentistry

**Published:** 1899-12

**Authors:** 


ARTICLE VII.

### PAINLESS DENTISTRY.

Dr. Clyde Payne has certainly contributed two valuable formulas towards "Painless Dentistry." He claims even better results for the painless excavation of "Sensitive Dentine," with his Obtundent, that with "Cataphoresis." *His Obtundent* is made of carbonate of potassium, glycerine, cocaine and carbolic acid. He makes a saturated solution of carbonate of potassium and glycerine, then a saturated solution of cocaine and carbolic acid. He mixes the two solutions on a warm glass slab. A flat bottle filled with hot water forms a good slab. The rubber dam is applied and the cavity is made as dry as possible by means of absorbent Japanese bibulous paper, absolute alcohol and hot blasts of air with the hot air syringe. A drop of the mixture is now placed in the cavity and the hot air blown on it as warm as the patient can bear. This is kept up for five minutes, after which the tooth can be excavated quite painlessly.

For the painless extraction of the teeth he has compounded the following "*Local Anæsthetic*:" Cocaine, 15 grains; Glycerine, 5 drachms; Nitro-glycerine, 1-10 grain; Morphia sulph., 1 grain; Atropia sulph., 1 grain; Carbolic acid, 3 drops, and distilled water sufficient to make 2 ounces. He says that Cocaine is the basis of all local anæsthetics and it is a dangerous drug to use, unless its physiological action is well understood, consequently the formula is devised with a view of obtaining the best results and of thwarting all dangerous issues. Hence, he says: "There is sufficient glycerine to localize the cocaine, holding it in apposition to the parts a sufficient length of time to complete the operation, and not so very long that it acts as an irritant and causes swelling. The nitro-glycerine is intended to stimulate the heart just in proportion as the cocaine may depress it. The sulphates of Morphia and Atropia overcome the after-pain. The carbolic acid is intended to preserve the solution. In patients who have a poor circulation sometimes there is a swelling with this formula, but it will be painless and will subside as soon as the anæsthetic, with which you have infiltrated the tissues, has become absorbed." The anæsthetic figures a one and a half per cent. solution of cocaine, and the solution or formula was used effectively *nine months* after it was compounded. Implantation can be made with this Local Anæsthetic painlessly. He uses it effectively by rubbing on the gum before adjusting a clamp which may impinge on the gum.

In using the hyperdermic syringe, the needle should be introduced into the gum tissue horizontally, that is, pointing towards the back of the mouth. It should never point against the bone. The needle should be forced in, in the direction stated, about a quarter of an inch, and the point of the finger of the left hand pressed on the gum where the point of the needle is. Pressure is now made on the piston, while the finger presses the gum. The anæsthetic is thus forced into the gum, which when effective assumes a whitish appearance as well as swelling up and forming a "weal," varying in size from a ten cent piece to a quarter of a dollar. Considerable force has to be used on the piston to make the anæsthetic infiltrate into the tissues and produce the anæsthesia. If the liquid is passed through the syringe, emptying it with little force, no effect will be attained; under such circumstances the gum will not be blanched and the weal will not be formed. It is, therefore, necessary to obtain the local anæsthesia that the liquid should be forced into the tissues slowly, forcibly and drop by drop.—Dental Office and Laboratory.

